# Modeled Scenario Projections for the Ebola Disease Outbreak Caused by Bundibugyo Virus, 2026

**DOI:** 10.15585/mmwr.mm7522e1

**Published:** 2026-06-11

**Authors:** Eric Q. Mooring, William T. Koval, Isobel Routledge, Inga Holmdahl, Kate Hudson, Guido España, Rebecca Kahn, Beau B. Bruce

**Affiliations:** ^1^Predict Division, Center for Forecasting and Outbreak Analytics, CDC; ^2^U.S. Public Health Service, Rockville, Maryland; ^3^Goldbelt Ltd., Washington, DC; ^4^Inform Division, Center for Forecasting and Outbreak Analytics, CDC; ^5^Hockley Systems, Ltd., Toronto, Ontario, Canada.

**Summary**

**What is already known about this topic?**

An outbreak of Bundibugyo virus disease (BVD), a type of Ebola disease, is currently ongoing, centered in the Ituri province of the Democratic Republic of the Congo (DRC).

**What is added by this report?**

CDC used a transmission model to project outbreak growth over 3 months, by using different assumptions about the number of deaths as of May 24, 2026, and by varying the percentages of persons with BVD who are successfully identified and isolated to prevent ongoing transmission. Assuming 50 cumulative deaths as of May 24, 2026, if 70% of patients were to enter isolation, only approximately one in 20 simulations projected an outbreak exceeding 10,000 cases within 3 months.

**What are the implications for public health practice?**

Large-scale, rapid public health action is needed to control the current outbreak, already the largest known BVD outbreak, from becoming one of the largest Ebola epidemics in history.

## Abstract

On May 15, 2026, the Ministries of Health in the Democratic Republic of the Congo and Uganda declared outbreaks of Bundibugyo virus disease (BVD), a type of Ebola disease. In response to reports of high numbers of suspected cases and deaths in these outbreaks, CDC simulated scenario projections to understand possible future morbidity and mortality. A branching process model with the capacity to model transmission-reducing nonpharmaceutical interventions was calibrated to three putative cumulative death counts and projected for four possible intervention scenarios ranging from poor (20%) to extremely high (95%) levels of isolation and treatment of symptomatic persons. The analysis suggested a plausible spillover event (i.e., the transmission of a virus from its natural animal reservoir to humans) in mid to late February 2026. With poor isolation levels of patients with BVD (20%) and no other interventions, the likelihood of an outbreak that exceeds 20,000 cases within 3 months is 65%. If, however a high proportion of patients were to enter isolation (70%), only a one in 20 chance is projected for an outbreak with ≥10,000 cases within 3 months. These results underscore the importance of strong public health interventions, because the current outbreak is already the largest known BVD outbreak and has the potential to quickly become one of the largest Ebola disease outbreaks ever recorded.

## Introduction

In May 2026, outbreaks of Bundibugyo virus disease (BVD) caused by species O*rthoebolavirus bundibugyoense*, a species of orthoebolavirus for which no approved vaccine or medication is currently available, were reported in the Ituri province in northeastern Democratic Republic of the Congo (DRC) and Uganda (*1*). As of June 2, 2026, a total of 378 confirmed cases (363 in DRC and 15 in Uganda) and 63 confirmed deaths (62 in DRC and one in Uganda) have been recorded (*2*). BVD causes a severe hemorrhagic fever. Bundibugyo virus is spread through direct contact with the body fluids of a person who is infected or has died from BVD. CDC modeled possible trajectories of the outbreak over 3 months. The models considered different assumptions about the cumulative number of deaths as of May 24, 2026, and different scenarios of public health intervention intensity, defined by the percentages of persons with BVD who are successfully isolated and therefore prevented from causing onward transmission.

## Methods

### Model Structure

CDC used a model to simulate BVD outbreaks. The model was adapted from one applied to previous viral hemorrhagic fever outbreaks, including a Marburg virus disease outbreak in Ethiopia in 2025. In this model, each simulated outbreak was initialized with one infected person, who represented the person first infected from a zoonotic source (a spillover event). This person infected a randomly generated number of additional persons based on assumptions about the basic reproductive number ([R_0_], the average number of persons in a susceptible population infected by an infected person). Any infected persons were added to the simulation at times selected according to the distribution of intervals from one infection to the next and, in turn, were able to cause further infections. This simulation, called a branching process, continued until either 1) none of the infected persons in a generation caused any secondary infections, indicating termination of the outbreak or 2) the simulation reached 5,000 deaths, indicating a very large and exponentially growing outbreak.

### Time Intervals

Intervals from infection to symptom onset, symptom onset to death, and symptom onset to recovery were held constant for all infections within each simulated outbreak but varied among simulated outbreaks. Simulated persons were never infectious before symptom onset or after recovery but could be infectious after death.

Assumptions about parameters were based on published estimates from previous Ebola outbreaks (Supplementary Box). Estimates specific to BVD were used when available.

### Model Calibration to Assumed Number of Deaths

Assumptions for the cumulative number of BVD deaths as of May 24, 2026, were based on publicly available situation reports from DRC.* The model was calibrated to three different numbers of cumulative deaths (50, 100, and 200) to account for uncertainty in the current number of deaths caused by BVD.

A simulated outbreak was compatible with the real-world outbreak if it reached the assumed number of cumulative deaths by May 24, 2026, and if the first death occurred on or before April 24, 2026. Outbreaks were simulated until 500 simulations met these criteria. The accepted 500 simulated outbreaks were used to infer when the outbreak began and served as the basis for scenario projections of interventions for each model calibration.

### Scenario Projections for Isolation

Four intervention scenarios were assessed for each calibration, each implementing a different level of isolation (i.e., percentage of symptomatic infected persons detected, isolated, and treated: 20% [poor], 50% [moderate], 70% [high], and 95% [extremely high]). The extremely high scenario was chosen to estimate a lower bound for transmission.

The intervention was assumed to start on May 24, 2026. On that day in each simulation, the designated percentage of symptomatic persons was selected to begin isolating, with an average delay of 2 days until isolation and treatment. The same percentage of persons who later developed signs or symptoms was selected to begin isolating, with an average delay of 2 days from symptom onset. Simulated persons in isolation were prevented from causing any onward transmission; the model implicitly assumed that isolated persons who died were safely buried (i.e., without washing or embalming and buried by trained teams using personal protective equipment).

Each simulation reported the cumulative number of cases and cumulative number of deaths from the date of spillover until August 22, which would be 90 days after interventions began. The percentages of simulations with <10,000, 10,000–19,999, and ≥20,000 cases and with <2,000, 2,000–3,999, and ≥4,000 deaths were calculated for all simulations in each scenario and separately for those with an R_0_ less than or equal to and greater than the median R_0_ value. The effective reproductive number (R_e_, the average number of onward infections per infectious person, accounting for immunity and public health interventions) was calculated for the preintervention and postintervention periods.

The branching process model was written in Rust (version 1.95.0; The Rust Development Team), and the model calibration and scenario projection pipeline was written in Python (version 3.14.4; Python Software Foundation). This activity was reviewed by CDC, deemed not research, and conducted consistent with applicable federal law and CDC policy.^†^

## Results

### Outbreak Size Projections and Inferred Spillover Date by Assumed Number of Deaths

**Assuming 50 deaths.** The model calibrated to 50 deaths estimated that the spillover event that triggered this outbreak most likely occurred on approximately February 19, 2026 (interquartile interval [IQI] = February 1–March 8). Assuming that 20% of infected persons were successfully isolated beginning May 24, 2026, projections showed ≥20,000 cumulative cases in 65% of simulations, ≥10,000 cumulative cases in 85% of simulations, and ≥4,000 cumulative deaths in 69% of simulations (Figure). Even with 50% of infected persons isolated, many simulations still projected these numbers of cases but were less likely to occur (17% of simulations projected ≥20,000 cases and 22% projected ≥4,000 deaths). At 70% isolation, projected outbreaks were much more likely to be smaller, but still of substantial size, with 94% of simulations projecting <10,000 cases and only 1% projecting ≥20,000 cases; similarly, at this isolation level, 90% of simulations projected <2,000 deaths and only 3% projected ≥4,000 deaths. R_e_ declined proportional to the percentage of infected persons successfully isolated (Supplementary Figure 1).

**FIGURE:**
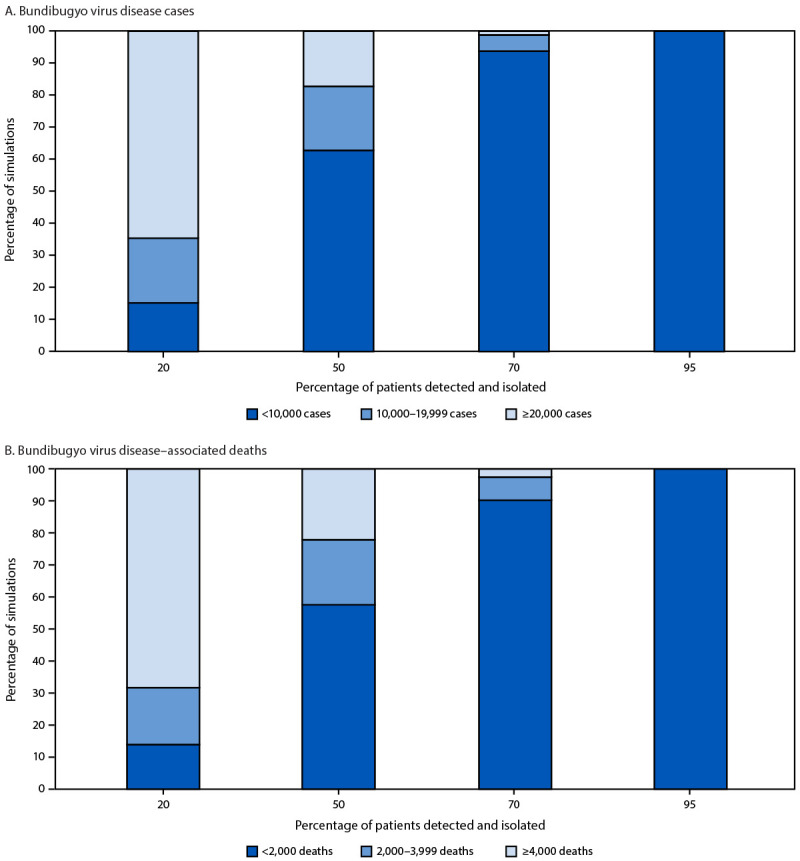
Percentage of simulated Bundibugyo virus disease outbreaks, by cumulative outbreak size category on August 22, 2026, as measured by cases (A) and deaths (B), and by percentage of simulated patients detected and isolated, using a branching process model,* 2026 * A branching process model is a type of infectious disease transmission model that starts with a single infection and simulates a transmission tree that represents an expanding outbreak. Simulations (500 for each vertical bar) assume 50 Bundibugyo virus disease–associated deaths as of May 24, 2026, and that the isolation intervention began that day.

**Assuming 100 deaths.** Assuming 100 cumulative deaths as of May 24, 2026, the inferred median spillover date was February 8, 2026 (IQI = January 21–February 27). Very large outbreaks were likely in the scenario in which only 20% of patients were isolated (76% of simulations projected ≥20,000 cases and 87% projected ≥4,000 deaths). In the scenario in which 70% of infected persons were isolated, 73% of simulations projected <2,000 cumulative deaths by August 22, 2026, and 10% projected ≥4,000 deaths (Supplementary Figure 2).

**Assuming 200 deaths.** Assuming 200 deaths by May 24, 2026, the calibrated model inferred a median spillover date of January 29, 2026 (IQI = January 9–February 18). The earlier spillover date would have generated a larger outbreak by the time interventions began; thus, even with 70% of infected persons isolated, 42% of simulations projected ≥10,000 cases by August 22, 2026.

### Sensitivity to Basic Reproductive Number

Simulated outbreaks with R_0_ values higher than the median R_0_ typically reached ≥10,000 cumulative cases and ≥2,000 cumulative deaths by August 22, 2026, in scenarios with ≤50% isolation, even assuming only 50 cumulative deaths by May 24. In the scenario with 70% of infected persons isolated and 50 assumed deaths by May 24, 2026, no simulations projected ≥2,000 deaths when R_0_ values were lower than the median R_0_, but 20% of simulations projected ≥2,000 deaths when R_0_ values exceeded the median (Supplementary Figure 3).

## Discussion

Model-based scenario projections of the current BVD outbreak suggest that if large-scale and sustained public health interventions are not rapidly implemented to reduce disease transmission, this outbreak could become as large as the 2014–2016 West Africa Ebola virus disease outbreak, which resulted in more than 28,000 cases and more than 11,000 deaths (*2*). Although the worst outcomes (higher numbers of cases and associated deaths) in these projections were less likely when a larger proportion of patients were identified, isolated, and treated, this outbreak could, within 3 months and under low-isolation scenarios, become the second largest Ebola outbreak in history. In light of this projected risk for a very large outbreak even if reasonably effective control measures are implemented, the public health response to control this outbreak will likely need to be of similar magnitude to the response for the 2014–2016 West Africa Ebola outbreak (*3*).

Even among simulations calibrated to only 50 deaths or those with a lower R_0_, very large outbreaks were still sometimes projected to occur, especially in scenarios without high levels of isolation. Calibrating the model to a larger number of deaths was approximately equivalent to assuming that interventions were implemented later in the outbreak. The results imply that intervening earlier in the outbreak would reduce the likelihood of worse outcomes.

The high probability of a large outbreak over a 3-month period primarily results from the large size of the outbreak at the time it was initially confirmed. This analysis did not provide evidence that R_0_ for this outbreak is unusually large.^§^ Time between Ebola outbreak onset and detection is positively correlated with overall outbreak size and duration (*4*).

CDC’s assessment that the risk to the general U.S. population is low (*5*) is not changed by this analysis. Despite the unprecedented size of the 2014–2016 West Africa Ebola epidemic, only two Ebola transmission events occurred in the United States. Those two infected persons were health care workers caring for a patient with Ebola who had traveled to the United States before enhanced screening, risk assessment, and health education measures were implemented at U.S. ports of entry (*6*). Both persons infected in the United States recovered.

### Limitations

The findings in this report are subject to at least five limitations. First, the true number of BVD deaths that occurred through May 24, 2026, is unknown. Some deaths from BVD might not have been confirmed; similarly, it is possible that other deaths might have been incorrectly attributed to BVD. Second, basic reproductive number estimates for Ebola disease vary widely across outbreaks. The true value of R_0_ for this outbreak might be higher or lower than the values used in this analysis. High-quality data on changes in the number of cases and deaths over time are essential to more precisely estimate R_0_. Third, changes in behavior that reduce risk for infection (e.g., avoiding contact with ill persons) were not included in the model and might help limit outbreak size. Fourth, the model did not account for transmission reductions attributable to an increase in the proportion of the population with infection-induced immunity. Given the population size of the communities where this outbreak is occurring, this limitation is unlikely to affect the validity of the projections over the time span and numerical ranges of cases presented in this analysis; however, the model could project unrealistically large outbreaks if applied to longer periods. Finally, the model did not include infection relapses after recovery (*7*). This limitation is unlikely to affect this analysis, but relapses could be important drivers of the course of the epidemic over a longer period.

### Implications for Public Health Practice

The current BVD outbreak is already the largest known BVD outbreak, and in scenarios with low percentages of isolated patients, could become one of the largest Ebola outbreaks ever documented. Urgent and sustained public health action is needed to prevent the outbreak from becoming as large as or larger than the 2014–2016 West Africa Ebola epidemic. This effort could require resources comparable in magnitude to the 2014–2016 Ebola response in West Africa. Rapid identification of cases, contact tracing, isolation and treatment of persons with BVD, community engagement, and use of safe and dignified burial for persons who die from BVD are necessary to control the outbreak.

## Footnotes

* The DRC national public health institute reported 10 confirmed deaths and 223 suspected deaths as of May 24, 2026. A subsequent report with data as of June 2, 2026, reported a cumulative total of 62 confirmed deaths in DRC.

† 45 C.F.R. part 46, 21 C.F.R. part 56; 42 U.S.C. Sect. 241(d); 5 U.S.C. Sect. 552a; 44 U.S.C. Sect. 552a; 44 U.S.C. Sect. 3501 et seq.

§ The calibrated values for R_0_ were nearly identical to the input assumption about R_0_. The median basic reproductive number R_0_ = 2.51 (IQI = 2.27−2.82) in the main analysis calibrated to 50 deaths.
